# Relationships of primary productivity with anuran abundance, richness, and community composition in tropical streams

**DOI:** 10.1371/journal.pone.0303886

**Published:** 2024-05-31

**Authors:** Jennifer A. Sheridan, Michael R. Kendrick

**Affiliations:** 1 Section of Amphibians and Reptiles, Carnegie Museum of Natural History, Pittsburgh, PA, United States of America; 2 South Carolina Department of Natural Resources, Marine Resources Research Institute, Charleston, SC, United States of America; Uppsala Universitet, SWEDEN

## Abstract

The relationship between primary productivity and diversity has been demonstrated across taxa and spatial scales, but for organisms with biphasic life cycles, little research has examined whether productivity of larval and adult environments influence each life stage independently, or whether productivity of one life stage’s environment outweighs the influence of the other. Experimental work demonstrates that tadpoles of stream-breeding anurans can exhibit a top-down influence on aquatic primary productivity (APP), but few studies have sought evidence of a bottom-up influence of primary productivity on anuran abundance, species richness and community composition, as seen in other organisms. We examined aquatic and terrestrial primary productivity in two forest types in Borneo, along with amphibian abundance, species richness, and community composition at larval and adult stages, to determine whether there is evidence for a bottom-up influence of APP on tadpole abundance and species richness across streams, and the relative importance of aquatic and terrestrial primary productivity on larval and adult phases of anurans. We predicted that adult richness, abundance, and community composition would be influenced by terrestrial primary productivity, but that tadpole richness, abundance, and community composition would be influenced by APP. Contrary to expectations, we did not find evidence that primary productivity, or variation thereof, predicts anuran richness at larval or adult stages. Further, no measure of primary productivity or its variation was a significant predictor of adult abundance, or of adult or tadpole community composition. For tadpoles, we found that in areas with low terrestrial primary productivity, abundance was positively related to APP, but in areas with high terrestrial primary productivity, abundance was negatively related to APP, suggesting a bottom-up influence of primary productivity on abundance in secondary forest, and a top-down influence of tadpoles on primary productivity in primary forest. Additional data are needed to better understand the ecological interactions between terrestrial primary productivity, aquatic primary productivity, and tadpole abundance.

## Introduction

The relationship between primary productivity and diversity has been demonstrated across taxonomic groups and spatial scales [1–5]. Primary productivity can be a fundamental driver of energy availability in a given system. While several theories exist to explain the link between primary productivity and biodiversity, one prominent theory is the more individuals hypothesis, which posits that higher energy availability will lead to more individuals in a given population, which in turn will foster species diversity [6,7]. Thus, primary productivity should be a positive predictor of both abundance and diversity, which has indeed been shown to be true across spatial scales and habitat types [8–10].

For organisms with biphasic life cycles, however, little research has examined whether primary productivity of the larval and adult environments influence each life stage independently, or whether primary productivity of one life stage’s environment outweighs the influence of the other. If the former were true, we should see strong correlation between the primary productivity of each life stage’s environment and the abundance and diversity of that life stage. In this scenario, there may or may not be a correlation between primary productivity of one life stage’s environment and the abundance and diversity of the organism’s other life stage. For example, frogs often have aquatic larvae and terrestrial adults. Aquatic primary productivity (APP) should predict larval (tadpole) abundance and diversity, terrestrial primary productivity (normalized differential vegetation index, NDVI, e.g.) should predict adult abundance and diversity, and adult abundance and diversity may or may not be related to APP. In the latter scenario, one measure of primary productivity (terrestrial or aquatic) would significantly predict abundance and diversity of both adult and larval anurans. In other words, we might see that NDVI is a significant predictor of both adult and larval abundance and diversity, regardless of APP, because 1) allochthonous inputs may support higher larval abundance, and 2) life history theory predicts that for species with high larval or juvenile mortality, population size (abundance) is more strongly influenced by conditions of the adult phase than the juvenile phase [11,12]. Thus, organisms with biphasic life cycles such as anurans present an interesting opportunity to test how primary productivity influences life stages, and thus helps quantify how expected changes in primary productivity (due to land use and climate change, for example) will influence abundance and diversity.

Experimental work demonstrates that for stream-breeding anurans, tadpoles can exhibit a top-down influence on aquatic primary productivity [13–15], but no study has yet examined whether there is evidence of a bottom-up influence of primary productivity on diversity, as has been seen in aquatic invertebrates [16] and terrestrial organisms [17–21]. In areas where amphibian larval development occurs largely in streams, there is the potential for stream (aquatic) primary productivity to have a bottom-up influence on tadpole abundance and diversity (adults choose more productive streams for egg laying, to provide offspring with sufficient resources for growth, or more productive streams have higher survival than streams with lower productivity), and that the terrestrial primary productivity will influence adult abundance and diversity [22–24]. Alternately, it’s possible that influences of terrestrial primary productivity on adult abundance and diversity, for example, outweigh impacts of primary productivity of the larval environment, and that larval abundance and diversity will be correlated with terrestrial primary productivity. No study has yet examined these influences for organisms such as anurans, so our aims were to a) determine whether there is evidence for a bottom-up influence of APP on tadpole abundance and diversity across streams, and b) determine the relative importance of aquatic and terrestrial primary productivity on both phases of anurans (larval and adult).

We examined aquatic and terrestrial primary productivity along streams in two forest types in Malaysian Borneo, along with amphibian abundance, diversity (species richness), and community composition at both larval and adult stages, in order to address our research aims. We predicted that adult species richness, abundance, and community composition would be influenced by terrestrial primary productivity more than by aquatic primary productivity, but that tadpole species richness, abundance, and community composition would be influenced by aquatic primary productivity more than terrestrial primary productivity. Ours is the first study to examine how these two measures of primary productivity influence overall community structure, abundance and species richness in amphibians.

## Materials and methods

### Study site

**Fig 1:**
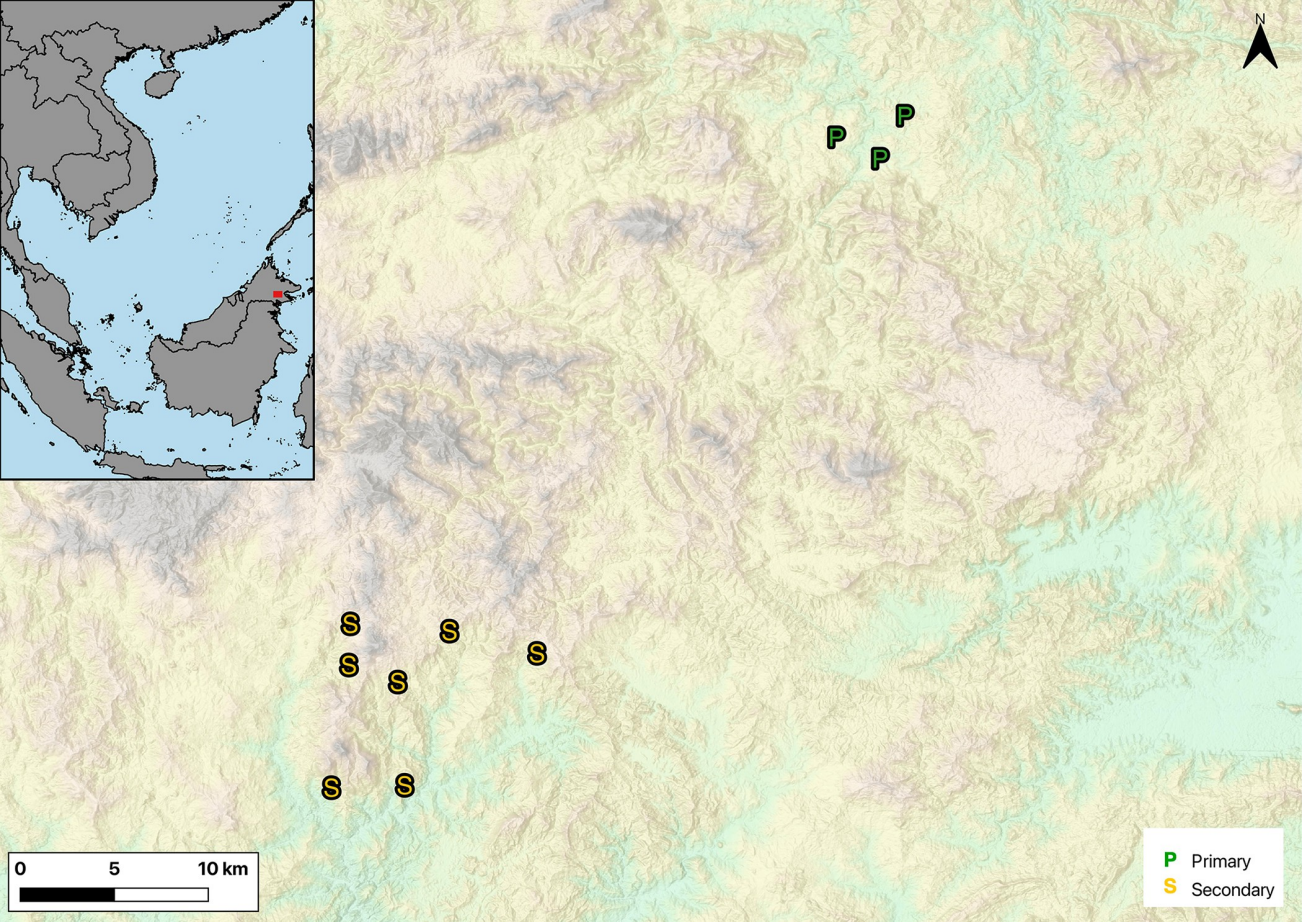
Locations of stream transects in primary (P; Danum Valley Conservation Area) and secondary (S; SAFE Project site) forest in Sabah, Malaysia, North Borneo. Base map republished from Esri under a CC BY license, with permission from Esri, original copyright 2020.

This study was conducted in Sabah, Malaysia (northern Borneo; Fig 1). With an area of 73,631 km^2^, Sabah has an array of land cover types (primary forest, secondary forest, agriculture, urban areas), consistent temperatures year-round, and all months receive at least 100 mm of rainfall. Sampling of primary (undisturbed) forest was in the Danum Valley Conservation Area (5°01″43′N 117°45″5′E), a lowland rainforest, designated as a conservation area of 4,380 km^2^ within the Sabah Foundation Forest Concession (Marsh & Greer, 1992) and one of Southeast Asia’s largest protected forests [25]. Sampling in fragmented forest occurred within a large-scale fragmentation experiment (SAFE project). The SAFE project [26] was established in 2011 by scientists (local and international), Sime Darby (the world’s largest palm oil producer), and Yayasan Sabah (Sabah forests’ governing body). Through this large collaborative initiative, a fixed area of forest in Borneo is being converted to oil palm plantations in a controlled manner, such that forest patches and riparian buffers of various sizes are left intact, enabling researchers to evaluate the impact of fragmentation.

### Amphibian surveys

We established one 200 m transect on each stream in 2012 for repeated surveys of adults and tadpoles, and these same transects were used each survey year. There were three streams in primary forest and seven streams in secondary forest (Fig 1).

Tadpoles were surveyed in primary forest and secondary forest in 2015 and 2017 (Table 1, S1 and S2 Tables in S1 File). Surveys were conducted diurnally using clear-bottomed basins pressed lightly into the water to search for tadpoles visually across the full width of the stream at 20 randomly chosen points along the transect, repeated six times each survey year, except when logistics (roads washed out, elephants prevented access to study site, etc.) reduced total surveys to 4 or 5 (Table 1). At each survey meter, we recorded stream width, species, and number of individuals. Identifications were based on examination and comparison with available museum specimens and are consistent with a recently published taxonomic key [27]. Of the 10 morphotypes, 5 were identified to the species level, 4 to the genus level, and 1 to family. Total survey area was calculated as the diameter of the survey basin (0.28 m) multiplied by stream width, totaled across all 20 survey meters for each survey day. Species richness was the total number of species observed during surveys in a given year, and abundance was the mean density (individuals/m^2^) of tadpoles on a given stream each year. One stream in secondary forest (120m) was not able to be surveyed in 2017 because water was never sufficiently clear enough to conduct surveys.

**Table 1:** Streams sampled in primary and secondary forest in Sabah, Malaysia for present study. Adults were sampled only in 2015. Number of tadpole surveys was six except where noted (in parentheses).

*Forest type*	*Stream*	*Tadpole survey years*
Primary	Palum Tambun	2015, 2017
Primary	Kalison	2015*, 2017
Primary	W6S5	2015, 2017
Secondary	0m	2015, 2017* (4)
Secondary	5m	2015, 2017 (5)
Secondary	15m	2015, 2017
Secondary	60m	2015, 2017 (5)
Secondary	120m	2015
Secondary	LFE	2015, 2017
Secondary	VJR	2015, 2017

*denotes missing aquatic primary productivity data.

Adults were surveyed in primary and secondary forest in 2015 (S3 Table in S1 File). The majority of amphibians in Borneo are stream-breeders [28], so stream surveys are the most efficient way to capture representative amphibian species richness in each study area. Visual encounter surveys were conducted by four people wading upstream [29], from shortly after dusk (18:45) until the full length of the transect had been surveyed, usually 2–3 hours. All individuals encountered were recorded for species, sex, and SVL, and released at point of capture. Voucher specimens and tissue samples were collected and deposited in the Sabah Muzium and North Carolina Museum of Natural Sciences. Every stream transect was searched four times in a given survey year during May–September. Species richness was estimated using the iNext function in R [30], set with an endpoint of 750 individuals, which is more than twice the highest number observed on any stream in a given year (S1 Fig). Abundance was the mean number of individual anurans observed per survey night each year.

For each stream, we measured canopy cover with a canopy densiometer every 10 m along the 200 m transect and took the mean of these as the measure of canopy cover for the stream. This was done once per survey year.

### Aquatic primary productivity

Aquatic primary productivity (APP) was measured along streams in primary and secondary forest in 2015 and 2017. One stream in each forest type has missing data from one survey year due to logistical complications (Table 1). We measured APP using the respirometer chamber method [31]. Streams in Borneo have rocky bottoms, and primary producers are attached to these rocks. Thus, primary production of the stream can be measured by placing individual rocks inside closed clear PVC chambers filled with water, with a dissolved oxygen meter inside each chamber. The dissolved oxygen meter measures the oxygen production by primary producers, which is recorded until the oxygen has increased by at least 2% or a minimum of 30 minutes, whichever occurs first. Respiration (the consumption of oxygen by microscopic organisms) is measured by placing a black bucket over the chamber, allowing us to measure the decrease in oxygen concentration in the absence of photosynthesis. Respiration is subtracted from production to yield net oxygen production, and the oxygen production is converted to mgC/m^2^/d, or aquatic primary productivity. APP was calculated as the mean of five or six rock measures per stream each year.

### Terrestrial primary productivity

We used Landsat 8 Level-2 normalized difference vegetation index (NDVI), a commonly used proxy for terrestrial primary productivity (TPP) in studies examining the relationship between TPP and animal diversity or species richness [32–34]. The NDVI is effectively a measure of how green an area is and can be generated from the near-infrared (NIR) and red bands of Landsat 8 images, providing a value from -1.0 to 1.0, calculated as (NIR—Red) / (NIR + Red). For each catchment, we calculated mean and standard deviation (variation) of NDVI (SD_NDVI) of each pixel from a total of 26 images (end date of surveys plus the previous 25 images, captured every 16 d, representing approximately 13 months of productivity). Pixels with cloud cover were omitted from analyses. Mean NDVI from 26 images was calculated for each pixel, omitting any that contained cloud cover, and catchment-level NDVI was calculated as the mean and SD of NDVI across all pixels for the sampling period.

### Statistical analyses

We tested for differences in primary productivity, species richness, and species abundance across forest types using Mann-Whitney U-tests. For variables that were measured on a given stream in two separate years, we calculated the mean value for each stream across years and used that value for comparisons across forest type. Adult species richness was estimated using the ‘iNext’ package [35] in R [30]. Because of how few individual tadpoles were observed on many streams, we used raw number of observed species as the measure of tadpole richness rather than an iNext estimate. Abundance was calculated as the mean number of individuals observed per survey night along the fixed transect length (adults) or mean density of tadpoles per survey meter (tadpoles). For adult community analyses we developed a Bray-Curtis dissimilarity matrix and, since tadpole community data were based on presence/absence information, we developed a Jaccard dissimilarity matrix for tadpole communities. Non-metric multidimensional scaling (NMDS) was used to visualize results and to test for the correlation of environmental factors with community structure using the envfit function in the ‘vegan’ package of R (Oksanen et al. 2020). Forest type and continuous variables with p<0.1 from the correlation tests were then included in permutational multivariate analysis of variance (PERMANOVA).

We also tested for correlates of environmental variables (canopy cover, mean NDVI, variation in NDVI (SD_NDVI), mean APP, and variation in APP (standard deviation in APP, SD_APP) with species richness and species abundance. For tadpoles, we examined the relationship between mean primary productivity (NDVI and APP), variation in productivity (SD_NDVI, SD_APP), and both richness and abundance with linear mixed models. For tadpole richness, we used the glmer function of the ‘lme4’ package of R [30,36] with a Poisson distribution, given that observed tadpole richness values were integers (discrete variables). For tadpole abundance, we used the lmer function of the ‘lme4’ package of R [30,36], given that abundance values were continuous. Predictor variables and year were fixed effects, and stream was a random effect. NDVI, APP, SD_NDVI, and SD_APP were normalized in R using “scale.” Because of strong correlation between NDVI and SD_NDVI, and between APP and SD_APP (S4 and S5 Tables in S1 File), no mixed models contained these pairs of variables. We tested combinations of year together with NDVI and APP or SD_NDVI and SD_APP for each response variable. For adults, because we had data from only a single survey year, we examined relationships between richness and abundance and the above-stated predictor variables using linear models in R. For tadpoles and adults, we used AIC to determine the best model(s) for a given response variable. All analyses were conducted in R [30].

## Results

### Primary productivity

Aquatic primary productivity and its variation (SD_APP) did not differ significantly between primary forest (APP: 112.3 ± 23.2; SD_APP: 63.6 ± 21.3) and secondary forest (APP: 83.0 ± 43.4; SD_APP: 52.3 ± 25.2; W = 16, p = 0.25 and W = 14, p = 0.49, respectively). Mean NDVI was higher (0.87 ± 0.005; W = 21, p = 0.02) and variation in NDVI was lower (0.02 ± 0.001; W = 0, p = 0.02) in primary forest than in secondary forest (NDVI: 0.82 ± 0.03; SD_NDVI: 0.06 ± 0.02; S1 Table in S1 File). Canopy cover, using means of values from 2015 and 2017 for each stream, was not a significant predictor of APP (F_1,6_ = 3.398, p = 0.115) nor of NDVI (F_1,6_ = 0.1694, p = 0.696).

### Species abundance, richness, and community composition

**Fig 2:**
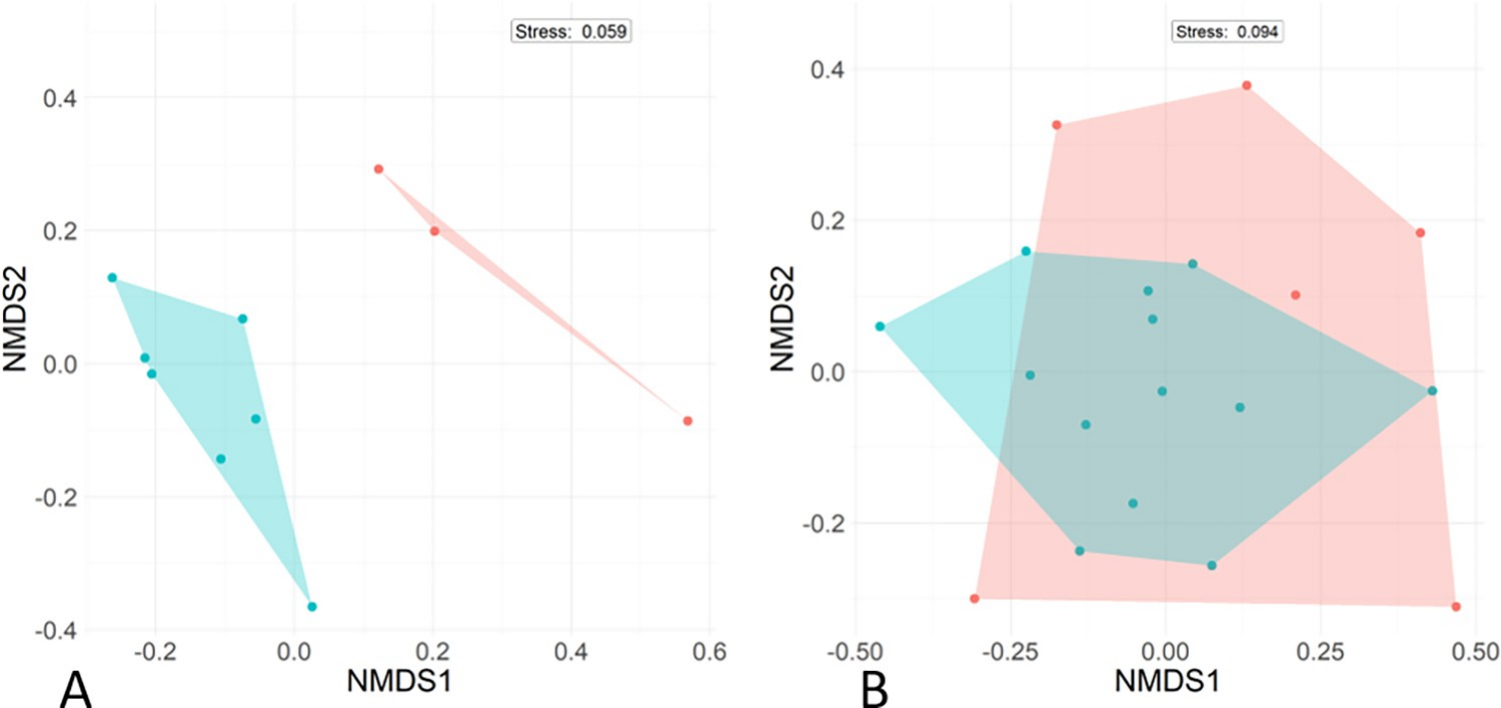
Results of non-metric multidimensional scaling (NMDS) comparison of A) adult and B) tadpole communities. Forest types are primary (undisturbed; P) indicated in red, and experimentally fragmented forest (secondary; S) indicated in blue.

Mean estimated adult species richness did not differ significantly between primary forest (19.2 ± 7.86) and secondary forest (14.3 ± 2.01; W = 15, p = 0.36), nor did adult abundance per survey night (primary forest: 32.7 ± 7.52; secondary forest: 47.5 ± 15.05; W = 4, p = 0.17). However, PERMANOVA indicated that adult community composition differed significantly between forest types (Pseudo-F_1,8_ = 4.91, R^2^ = 0.38, p < 0.01; Fig 2A).

Tadpole species richness did not differ between forest types (primary forest: 2.0 ± 1.00; secondary forest: 3.7 ± 1.38; W = 5, p = 0.24), nor did tadpole abundance (density per m^2^ in primary forest: 0.11 ± 16; secondary forest: 0.16 ± 0.17; W = 2, p = 0.07; S1 Table in S1 File). Additionally, even though there is strong overlap in the NMDS figure, PERMANOVA indicated that tadpole community composition differed significantly between forest types (Pseudo-F_1,18_ = 3.34, R^2^ = 0.16, p = 0.01; Fig 2B).

### Predictors of species abundance, richness, and community composition

**Fig 3:**
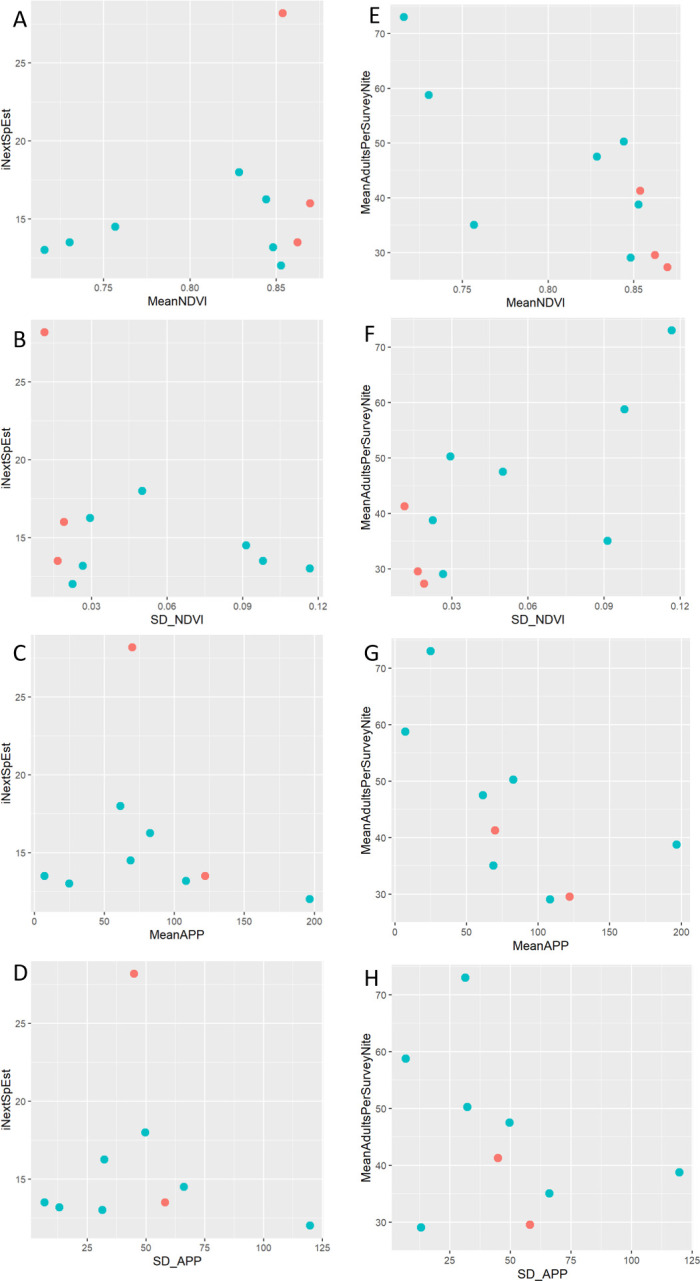
Primary productivity (NDVI, SD_NDVI, MeanAPP, and SD_APP) as it relates to species richness (iNextSpEst; A–D), and abundance (MeanIndPerSurveyNight; E–H) of adult frogs along study streams in Sabah, Malaysia. Red dots represent undisturbed (primary) forest and blue dots represent experimentally fragmented (secondary) forest.

**Fig 4:**
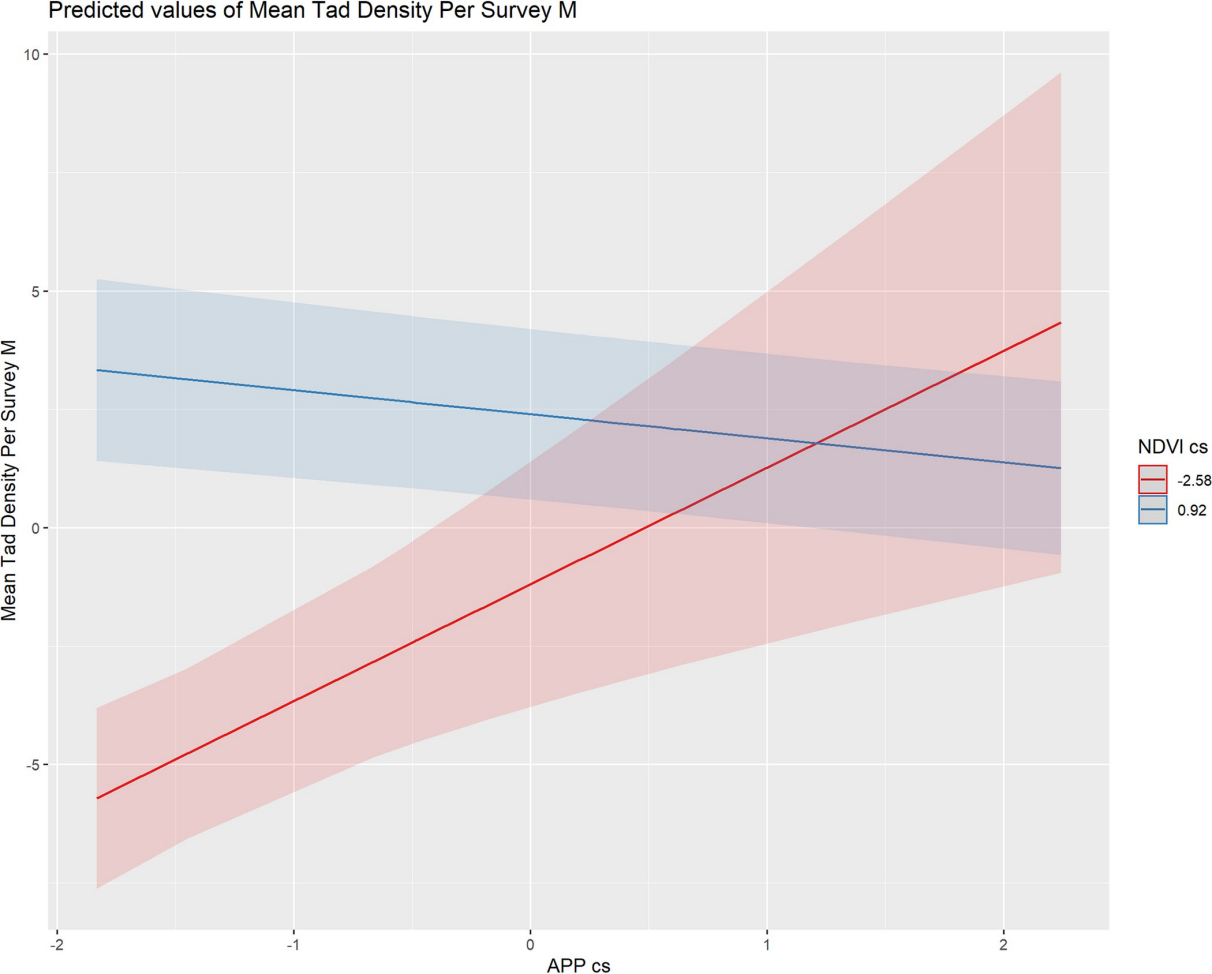
Predicted tadpole abundance (mean tadpole density per survey meter) for low (red) and high (blue) levels of scaled values of NDVI, with respect to scaled values of APP.

Adult species richness was not predicted by any measure of productivity (S6 Table in S1 File, Fig 3A–3D). Adult abundance was negatively related to NDVI and positively to sdNDVI, but was not related to APP nor sdAPP (Fig 3E–3H; S8 Table in S1 File). Tadpole richness was not predicted by any of our tested variables (S7 Table in S1 File) but tadpole abundance was significantly predicted by an interaction between NDVI and APP (S9 Table in S1 File), such that at low levels of NDVI (secondary forest), there are more tadpoles at high levels of APP than at low levels of APP, while at high levels of NDVI (primary forest), there are more tadpoles at low levels of APP than at high levels of APP (S10 Table in S1 File; Fig 4).

Species richness was not predicted by abundance for adults (R^2^ = 0.02, F_1,12_ = 0.21, p = 0.66) or tadpoles (R^2^ = 0.14, F_1,18_ = 3.045, p = 0.098). Additionally, no environmental variable was a significant predictor of community composition of adults or tadpoles (S11 and S12 Tables in S1 File).

## Discussion

Consistent with previous literature [37,38], but in contrast to some studies [39,40], terrestrial primary productivity (NDVI) was higher and more stable (variation was lower) [38,39] in primary forest than in secondary forest. Contrary to previous results showing how forest age can influence aquatic primary productivity (APP) [41], our study found no difference in APP or its variation between streams in the two forest types. Although NDVI and APP were positively correlated, variation in NDVI only explained 23% of the variance in APP, highlighting the likely role of in-stream processes mediating landscape controls on stream ecosystem function [42].

While abundance of grazers tend to limit primary productivity of periphyton [43,44], the relative importance of top-down and bottom-up controls can be variable [45]. The relationships among tadpole abundance, NDVI, and APP presented in this study indicate that terrestrial processes may be shaping the extent of top-down and bottom-up control in these systems. Our data indicate that tadpole abundance is predicted by an interaction between NDVI and APP, and that at high levels of NDVI such as are found in primary forest, tadpoles have a top-down influence on APP (higher tadpole abundance is associated with low levels of APP), supporting experimental work from tropical streams elsewhere [13,46], as well as from pond and lake systems [47–49]. However, at low levels of NDVI, such as those seen in secondary forest, we found that higher tadpole abundance is associated with high levels of APP, indicating a bottom-up influence of APP on tadpole abundance. Additional data are needed to better understand the ecological interactions between terrestrial productivity, aquatic primary productivity, and tadpole abundance, but it is notable that ours is the first study to provide evidence supporting a shift from top-down to bottom-up processes for stream tadpoles between primary and secondary forests. The apparent top-down impact of tadpoles on APP in areas with high NDVI may indicate that amphibians are a key consumer guild in tropical primary forest streams.

NDVI may be used as an indicator for allochthonous inputs into these streams where higher NDVI, a proxy for terrestrial productivity, may have higher allochthonous subsidies to streams. Many tadpoles feed on both autochthonous and allochthonous material [23] and these allochthonous subsidies may support increased secondary production of anurans, exacerbating top-down influence on autochthonous production. These findings are consistent with previous studies showing that allochthonous subsidies can exacerbate top-down controls [50,51] and alter food web dynamics [52]. In areas of low NDVI, for instance, the positive relationship between tadpole abundance and APP suggests a bottom-up control on tadpole abundance. In areas of high NDVI, however, the negative relationship between tadpole abundance and APP suggests that tadpoles may exert top-down influence on APP.

Our results show that both in-stream APP and terrestrial NDVI were important factors in determining tadpole abundance, highlighting the importance of both in-stream and terrestrial processes. Tropical anurans can have a range of feeding strategies including both grazers and detritivores, with both autochthonous and allochthonous inputs supporting anuran production and many species feeding from both pathways [53–55]. In our study, sites with higher terrestrial primary productivity (as NDVI) did not show higher levels of canopy cover but did likely have higher rates of allochthonous inputs due to higher primary production in the surrounding forest. Furthermore, additional factors such as nutrient availability and disturbance can shift the baseline periphyton availability, and thus modulate grazer abundance through bottom-up processes [56].

Our observed tadpole richness (n = 1–6) was similar to tadpole surveys in other parts of Sabah [n = 8; 57], but lower than in other areas of Borneo [n = 17 and 29, respectively; 58,59]. We did not find support for the more individuals hypothesis [6,7] to explain adult or tadpole species richness. Additionally, contrary to expectations [18,20,21], we did not find evidence that primary productivity, or variation in productivity, predicts anuran species richness at either the tadpole or adult stage. Further, no measure of productivity or its variation was a significant predictor of adult abundance, or of adult or tadpole community composition. It is interesting that while tadpole abundance is predicted by an interaction between NDVI and APP, and tadpole species richness is predicted by abundance, we did not find evidence that tadpole species richness is directly predicted by NDVI or APP or their interaction. Future work including multiple sampling methods to gain a more complete picture of tadpole species richness on any stream may reveal more details of how both terrestrial and aquatic primary productivity relate to tadpole species richness and abundance, but our work provides an important starting point for such questions.

The lack of relationship between productivity and species richness of both adults and tadpoles in our study was, in some ways, surprising, given the multiple studies that report a positive relationship between productivity and amphibian diversity or species richness, at regional and global scales [17–21,60]. The majority of studies examining the relationship between primary productivity and amphibian diversity or species richness either examine the relationship across a variety of protected areas [regional studies; 17,18,60] or utilize coarse-grain global distribution maps [global studies; 18,19–21]. Regional studies examining the relationship between productivity and diversity or species richness are not generally designed to detect differences in the productivity-diversity relationship across land use or forest types. The lack of a productivity-diversity relationship in our study system in Borneo, at either the adult or larval stage, may indicate that local factors [e.g., disturbance regime; 16] may play an important role and that land use can have an important influence on the nature of productivity-diversity relationships. We recommend further investigations into these relationships across an array of forest types to determine whether fragmentation or disturbance indeed alter the predicted relationship, or whether amphibians in our study system do not adhere to the predicted relationship between primary productivity and species richness.

The biphasic life cycle of anurans makes them susceptible to shifting environmental conditions in both aquatic and terrestrial environments, with land-use changes not only influencing the productivity in both phases of the system [61], but also the disturbance regime [62] and biogeochemical processes [63] that help control ecological processes in these ecosystems. We recommend additional sampling of amphibian adults and larvae in conjunction with aquatic and terrestrial primary productivity, across an array of land-use types, to better define the relationships between these variables.

## Supporting information

S1 Fig Species accumulation (solid lines) and rarefaction (dashed lines) curves for each surveyed stream in 2015, calculated using iNEXT in R. Shading represents 95% confidence intervals. (JPG)

S1 File Contains all supporting tables. (DOCX)
