# Supplementary material for: Relationships of primary productivity with anuran abundance, richness, and community composition in tropical streams
Source: PLoS One. 2024 May 31;19(5):e0303886. doi: 10.1371/journal.pone.0303886 (PMC11142703; doi:10.1371/journal.pone.0303886)
Supplement: S1 File — (DOCX) [file pone.0303886.s002.docx]

Table S1. Summary data of streams in primary (Danum) and recently fragmented (SAFE) forest in Sabah, Malaysian Borneo.

|  |  |  |  |  |  |  |  | Adults | | | | Tadpoles | | | |
| --- | --- | --- | --- | --- | --- | --- | --- | --- | --- | --- | --- | --- | --- | --- | --- |
| Year | P/S | Stream | Mean  Canopy | Mean  NDVI | SD NDVI | Mean APP | SD APP | n | nSp | iNext SpEst | Mean Ind/  Survey | n | nSp | Mean Density/m^2^ | Mean Density/200m^2^ |
| 2015 | P | Kalison | 84.93 | 0.87 | 0.02 | NA | NA | 4 | 14 | 15.98 | 27.25 | 6 | 2 | 0.01 | 2.81 |
| 2015 | P | Palum Tambun | 79.22 | 0.86 | 0.02 | 122.10 | 58.26 | 4 | 12 | 13.49 | 29.50 | 6 | 1 | 0.03 | 6.09 |
| 2015 | P | W6S5 | 89.29 | 0.85 | 0.01 | 70.05 | 45.07 | 4 | 16 | 28.18 | 41.25 | 6 | 3 | 0.30 | 60.53 |
| 2017 | P | Kalison | 78.60 | 0.87 | 0.02 | 102.77 | 68.20 | NA | NA | NA | NA | 6 | 4 | 0.00 | 0.00 |
| 2017 | P | Palum Tambun | 82.41 | 0.88 | 0.02 | 155.41 | 106.21 | NA | NA | NA | NA | 6 | 1 | 0.00 | 0.00 |
| 2017 | P | W6S5 | 87.09 | 0.87 | 0.02 | 120.58 | 35.64 | NA | NA | NA | NA | 6 | 2 | 0.00 | 0.00 |
| 2015 | S | 0M | 75.70 | 0.76 | 0.09 | 68.87 | 66.28 | 4 | 13 | 14.49 | 35.00 | 6 | 3 | 0.18 | 36.25 |
| 2015 | S | 5M | 91.13 | 0.73 | 0.10 | 7.26 | 6.88 | 4 | 11 | 13.49 | 58.75 | 6 | 3 | 0.06 | 11.06 |
| 2015 | S | 15M | 60.59 | 0.83 | 0.05 | 61.56 | 49.73 | 4 | 12 | 17.98 | 47.50 | 6 | 2 | 0.02 | 4.49 |
| 2015 | S | 60M | 78.16 | 0.72 | 0.12 | 25.11 | 31.46 | 4 | 12 | 13.00 | 73.00 | 6 | 6 | 0.20 | 40.31 |
| 2015 | S | 120M | 53.55 | 0.85 | 0.02 | 196.76 | 119.86 | 4 | 11 | 11.99 | 38.75 | 6 | 4 | 0.50 | 100.67 |
| 2015 | S | LFE | 80.05 | 0.84 | 0.03 | 82.79 | 32.41 | 4 | 14 | 16.24 | 50.25 | 6 | 5 | 0.16 | 31.10 |
| 2015 | S | VJR | 53.19 | 0.85 | 0.03 | 108.49 | 13.21 | 4 | 13 | 13.17 | 29.00 | 6 | 3 | 0.01 | 2.78 |
| 2017 | S | 0M | 86.15 | 0.85 | 0.04 | NA | NA | NA | NA | NA | NA | 4 | 3 | 0.05 | 9.96 |
| 2017 | S | 5M | 88.21 | 0.85 | 0.05 | 24.94 | 34.67 | NA | NA | NA | NA | 5 | 5 | 6.39 | 1278.75 |
| 2017 | S | 15M | 68.84 | 0.84 | 0.05 | 118.32 | 106.49 | NA | NA | NA | NA | 6 | 3 | 0.73 | 146.66 |
| 2017 | S | 60M | 84.41 | 0.85 | 0.06 | 81.66 | 36.87 | NA | NA | NA | NA | 5 | 4 | 7.07 | 1413.89 |
| 2017 | S | 120M | 56.83 | 0.81 | 0.07 | 111.88 | 51.72 | NA | NA | NA | NA | NA | NA | NA | NA |
| 2017 | S | LFE | 87.71 | 0.86 | 0.03 | 104.04 | 27.35 | NA | NA | NA | NA | 6 | 4 | 0.34 | 67.53 |
| 2017 | S | VJR | 71.25 | 0.86 | 0.05 | 102.05 | 88.94 | NA | NA | NA | NA | 6 | 2 | 0.03 | 5.62 |

Table S2. Tadpole species observed on all surveyed streams in primary (Danum: Kalison, Palum Tambun, W6S5) and recently fragmented (SAFE: 0m, 5m, 15m, 60m, 120m, LFE, VJR) forest in Sabah, Malaysian Borneo.

| **Species** | **StreamYear** |
| --- | --- |
| Bufonidae |  |
| *Ansonia spinulifer* | 0m2015, 5m2015, 60m2015, 120m2015, LFE2015,  0m2017, 5m2017, 120m2017, LFE2017, VJR2017 |
| Microhylidae |  |
| *Microhyla sp.* | 5m2017, 15m2017, W6S52017 |
| Megophryidae |  |
| *Leptobrachium abbotti* | 0m2015, 5m2015, 60m2015, LFE2015, VJR2015,  0m2017, 5m2017, 15m2017, 60m2017, LFE2017, Kal2017, W6S52017 |
| *Leptolalax sp.* | Kal2017 |
| Ranidae |  |
| *Chalcorana megalonesa* | 0m2015, 60m2015, 120m2015, LFE2015, W6S52015,  PT2017 |
| *Limnonectes sp.* | 15m2015,  5m2017, 60m2017, PT2017 |
| *Meristogenys orphnocnemis* | 5m2015, 60m2015, 120m2015, LFE2015, VJR2015, Kal2015,  0m2017, 5m2017, 15m2017, 60m2017, LFE2017, VJR2017 |
| *Staurois sp.* | 60m2015, W6S52015,  Kal2017 |
| Rhacophoridae |  |
| *Rhacophorus gauni* | 15m2015, 60m2015, 120m2015, LFE2015, VJR2015, Kal2015, PT2015, W6S52015, LFE2017 |
| *Rhacophorus sp.* | 60m2017 |

Table S3. Adult species observed on streams at Danum Valley (Kalison, Palum Tambun, W6S5) and SAFE (0m, 5m, 15m, 60m, 120m, LFE, VJR) in 2015, Sabah, Malaysian Borneo.

| **Species** | **K** | **PT** | **W6S5** | **0m** | **5m** | **15m** | **60m** | **120m** | **LFE** | **VJR** |
| --- | --- | --- | --- | --- | --- | --- | --- | --- | --- | --- |
| Bufonidae |  |  |  |  |  |  |  |  |  |  |
| *Ansonia leptopus* |  | x | x |  |  |  |  |  |  |  |
| *Ansonia spinulifer* | x |  | x | x | x |  | x |  | x |  |
| *Phrynoidis juxtasper* |  | x | x | x | x | x | x | x | x | x |
| *Rentapia hosii* | x | x |  |  |  |  |  |  |  |  |
| Ceratobatrachidae |  |  |  |  |  |  |  |  |  |  |
| *Alcalus baluensis* | x | x |  |  |  |  |  |  |  |  |
| Dicroglossidae |  |  |  |  |  |  |  |  |  |  |
| *Limnonectes finchi* | x |  | x | x | x | x | x |  | x |  |
| *Limnonectes kuhlii* | x | x | x | x | x | x | x | x | x | x |
| *Limnonectes leporinus* | x | x | x | x |  | x |  | x | x | x |
| *Limnonectes pa???* |  |  | x |  |  |  |  |  |  |  |
| *Occidozyga laevis* | x |  |  |  |  |  |  |  |  |  |
| Megophryidae |  |  |  |  |  |  |  |  |  |  |
| *Leptobrachium abbotti* |  |  | x |  |  |  |  |  | x |  |
| *Leptolalax dringi* | x |  | x | x | x |  | x | x | x | x |
| *Megophrys nasuta* |  |  |  |  | x |  |  |  |  |  |
| Microhylidae |  |  |  |  |  |  |  |  |  |  |
| *Chaperina fusca* |  | x |  |  |  | x |  |  |  |  |
| Ranidae |  |  |  |  |  |  |  |  |  |  |
| *Chalcorana megalonesa* | x | x | x | x | x | x | x |  | x | x |
| *Hylarana erythraea* |  |  |  |  |  | x |  |  |  |  |
| *Meristogenys orphnocnemis* | x | x | x | x | x | x | x | x | x | x |
| *Odorrana hosii* |  |  |  |  |  |  | x |  |  |  |
| *Pulchrana picturata* | x | x | x | x |  |  | x | x | x | x |
| *Staurois guttatus* | x |  | x | x | x | x | x | x | x | x |
| *Staurois latopalmatus* |  |  |  | x | x | x | x | x | x | x |
| Rhacophoridae |  |  |  |  |  |  |  |  |  |  |
| *Philautus hosii* | x |  | x |  |  |  |  |  |  |  |
| *Philautus tectus* |  | x |  |  |  |  |  |  |  |  |
| *Polypedates colletti* |  |  |  |  |  |  |  | x |  |  |
| *Polypedates otilophus* |  |  |  |  |  |  |  |  |  | x |
| *Rhacophorus gauni* | x | x | x | x | x | x | x | x | x | x |
| *Rhacophorus pardalis* |  |  | x | x |  | x |  | x | x | x |

**Correlation of environmental variables.**

Table S4. Pearson’s product-moment correlation for environmental variables measured in 2015. Bolded values are those that were significant after Bonferroni correction (p < 0.0167).

| Variables | T | df | P | cor |
| --- | --- | --- | --- | --- |
| **NDVI, SD_NDVI** | **-17.261** | **8** | **< 0.001** | **-0.986** |
| NDVI, APP | 2.825 | 7 | 0.026 | 0.730 |
| NDVI, SD_APP | 0.988 | 7 | 0.356 | 0.350 |
| **APP, SD_APP** | **3.146** | **7** | **0.016** | **0.765** |
| APP, SD_NDVI | -2.628 | 7 | 0.034 | -0.705 |
| SD_NDVI, SD_APP | -0.877 | 7 | 0.410 | -0.315 |

Table S5. Pearson’s product-moment correlation for environmental variables measured in 2017. Bolded values are those that were significant after Bonferroni correction (p < 0.0167).

| Variables | T | df | P | cor |
| --- | --- | --- | --- | --- |
| **NDVI, SD_NDVI** | **-4.978** | **8** | **0.001** | **-0.869** |
| NDVI, APP | 0.671 | 7 | 0.524 | 0.246 |
| NDVI, SD_APP | -0.282 | 7 | 0.786 | 0.106 |
| APP, SD_APP | 1.808 | 7 | 0.113 | 0.564 |
| APP, SD_NDVI | -1.184 | 7 | 0.275 | -0.409 |
| SD_NDVI, SD_APP | -0.280 | 7 | 0.788 | -0.105 |

**Species richness.**

NDVI was strongly negatively correlated with sdNDVI, so no models contain both of these variables. Similarly, APP was strongly positively correlated with sdAPP, so no models contain both of these variables. Thus, we tested mixed models of the remaining possible combinations of variables.

Table S6. Fixed effects estimates and p-values from models testing for the effects of terrestrial (NDVI, sdNDVI) and aquatic (APP, sdAPP) productivity on adult species richness. Example model structure: Model 1a: *lm(iNextSpEst~NDVI)*

| Model | NDVI | sdNDVI | APP | sdAPP | p-value |
| --- | --- | --- | --- | --- | --- |
| 1a | 1.409 |  |  |  | 0.40 |
| 2a |  | -1.711 |  |  | 0.30 |
| 3a |  |  | -0.9879 |  | 0.61 |
| 4a |  |  |  | -0.4761 | 0.81 |

Table S7*.* Fixed effects estimates and p-values from top model testing for the effects of terrestrial (NDVI, sdNDVI) and aquatic (APP, sdAPP) productivity on tadpole species richness. Model structure: *lmer(nSpTads~NDVI + APP + Year +(1|Stream))*.

| NDVI | APP | Year | p-value |
| --- | --- | --- | --- |
| -0.0258 |  |  | 0.96 |
|  | -0.5051 |  | 0.30 |
|  |  | -0.1100 | 0.78 |

**Abundance.**

Table S8. Fixed effects estimates and p-values from models testing for the effects of terrestrial (NDVI, sdNDVI) and aquatic (APP, sdAPP) productivity on adult abundance. Example model structure: Model 1a: *lm(MeanAdultsPerSurveyNite~NDVI)*

| Model | NDVI | sdNDVI | APP | sdAPP | p-value |
| --- | --- | --- | --- | --- | --- |
| 1a | -10.969 |  |  |  | 0.013 |
| 2a |  | 10.605 |  |  | 0.018 |
| 3a |  |  | -9.504 |  | 0.053 |
| 4a |  |  |  | -4.955 | 0.364 |

Table S9. Fixed effects estimates from models testing for the effects of terrestrial (NDVI, sdNDVI) and aquatic (APP, sdAPP) productivity on tadpole abundance (mean tadpole density). Bold values indicate p < 0.05. Example model structure, Model 5t: *lmer(MeanTadDensity~NDVI * APP + Year +(1|Stream))*.

| Model | NDVI | sdNDVI | APP | sdAPP | NDVI*APP | sdNDVI*APP | Year | ΔAIC |
| --- | --- | --- | --- | --- | --- | --- | --- | --- |
| 5t | **1.026** |  | 0.273 |  | -**0.849** |  | -0.024 | 0 |
| 6t | **2.473** |  | -0.727 |  |  |  | 0.033 | 5.25 |
| 7t |  | 0.192 |  | -0.526 |  | -0.074 | 0.898 | 14.04 |
| 8t |  | 0.223 |  | -0.511 |  |  | 0.886 | 13.08 |

Table S10. Fixed effect estimates from the GLMM quantifying changes in scaled NDVI (NDVI), scaled APP (APP), their interaction (NDVI:APP), and year with tadpole abundance (MeanTadDensity) on Borneo streams (Model 5t, above).

*Estimate Std. Error df t-value p-value*

*(Intercept)* 50.5544 129.895 3.997 0.389 0.717

NDVI 1.0262 0.3485 4.409 2.944 0.037

APP 0.2727 0.2465 4.401 1.107 0.325

Year -0.0244 0.0644 3.996 -0.378 0.725

NDVI:APP -0.8493 0.1883 4.390 -4.509 **0.009**

*Model structure: lmer(MeanTadDensity~NDVI * APP +Year + (1|Stream))*

**Community composition.**

Table S11. Vectors from ordination analyses examining the relationship between adult community composition (NMDS1 and NMDS2) and terrestrial productivity (NDVI), variation in terrestrial productivity (sdNDVI), aquatic primary productivity (APP), and variation in aquatic primary productivity (sdAPP).

|  | NMDS1 | NMDS2 | r^2^ | Pr(>r) |
| --- | --- | --- | --- | --- |
| Canopy Cover | 0.14269 | 0.98977 | 0.4681 | 0.141 |
| NDVI | 0.99928 | -0.03781 | 0.2614 | 0.415 |
| sdNDVI | -0.96480 | -0.26300 | 0.2692 | 0.408 |
| APP | 0.75664 | -0.65383 | 0.1334 | 0.633 |
| sdAPP | 0.60024 | -0.79982 | 0.0785 | 0.746 |

Table S12. Vectors from ordination analyses examining the relationship between tadpole community composition (NMDS1 and NMDS2) and terrestrial productivity (NDVI), variation in terrestrial productivity (sdNDVI), aquatic primary productivity (APP), and variation in aquatic primary productivity (sdAPP).

|  | NMDS1 | NMDS2 | r^2^ | Pr(>r) |
| --- | --- | --- | --- | --- |
| Canopy Cover | 0.04595 | -0.99894 | 0.0145 | 0.897 |
| NDVI | 0.84153 | -0.54021 | 0.0925 | 0.495 |
| sdNDVI | -0.95227 | -0.30525 | 0.1368 | 0.338 |
| APP | 0.90173 | -0.43230 | 0.0222 | 0.870 |
| sdAPP | 0.99968 | 0.02547 | 0.0112 | 0.918 |
